# Factors influencing medical students’ decisions on specialty training in radiology at King Khalid University, Abha, Saudi Arabia: A cross-sectional study

**DOI:** 10.1097/MD.0000000000049096

**Published:** 2026-05-29

**Authors:** Hassan Ali Almubarak, Abdulrahman Ali H. Al Mesmer Almaymoni, Waad Saber Z. Alharethi, Ohoud Hussain M. Moalwi, Lamyaa Safar A. Almathahibi, Reema Saleh A. Alqahtani, Ahmed Ali Khuzayyim, Hanan Delem M. Almoghamer, Fatimah Ahmed H. Badawi, Wajan Ahmed A. Alshahrani, Lujain Adel A. Daghriri, Khalid Abdulrahman A. Almehery, Awad Alsamghan, Syed Esam Mahmood

**Affiliations:** a Department of Medicine, College of Medicine, King Khalid University, Abha, Aseer, Saudi Arabia; b College of Medicine, King Khalid University, Abha, Aseer, Saudi Arabia; c Department of Family and community Medicine, College of Medicine, King Khalid University, Abha, Aseer, Saudi Arabia.

**Keywords:** medical students, Radiology, Saudi Arabia, selection criteria, specialty

## Abstract

Radiology is an essential medical specialty, yet the decision to pursue it is shaped by several factors, including educational exposure, perceived workload, and patient interaction. This study explores the factors influencing medical students and interns at King Khalid University (KKU), Abha, Saudi Arabia, in selecting radiology as a specialty. A cross-sectional survey was conducted among 380 medical students and interns at KKU. The participants were asked about their demographic background, exposure to radiology, and the factors influencing their specialty choices. Data analysis was performed using Statistical Package of Social Science Software version 23, with Chi-square and Fisher’s exact tests applied to assess associations between variables. A *P* value of <.05 was considered statistically significant. Of the 380 participants, 62.9% had some exposure to radiology, and 39.2% considered radiology as a future specialty. The most commonly cited reasons for choosing radiology included its diversity (68.2%), minimal patient interaction, and the interesting content (63.4%). Conversely, radiation exposure (78.7%) and choosing another specialty (53.4%) were the main reasons for not pursuing radiology. A significant association was found between exposure to radiology and the likelihood of choosing it as a specialty (*P* < .001). Technological advancements in Radiology appeal to many students, but concerns about radiation exposure and patient interaction deter others. Addressing these concerns through early educational exposure and mentorship could increase interest in the specialty. Further longitudinal research across multiple institutions is needed to gain deeper insights.

## 1. Introduction

The choice of medical specialty profoundly influences the future healthcare workforce, impacting the availability of medical services and the organization of healthcare systems. In Saudi Arabia, where rapid population growth necessitates comprehensive healthcare services, understanding the factors guiding medical students in specialty selection is critical. Specialty choice occurs primarily during the final year or internship, a high-pressure period where students must reconcile personal and professional aspirations.^[1,2]^

Existing literature indicates that a majority of medical students lean toward clinical specialties, often neglecting preclinical and paraclinical fields.^[3]^ This trend poses challenges for medical faculties and can create imbalances in the distribution of healthcare professionals across specialties. Factors influencing the choice of specialty decisions include family commitments, potential income, and the proximity of healthcare facilities, particularly affecting students from rural backgrounds.^[4]^

In radiology, the implications of specialty choice are pronounced. Radiologists require extensive training, often completing fellowships postresidency. Although demand for imaging services is increasing: evidenced by a 5.5% annual rise in relative value units—radiologist growth in the United States is stagnating at 2% annually.^[5]^ This discrepancy underscores an urgent need for reform in radiology training programs to attract and retain specialists.

Furthermore, gender disparities are evident in radiology, with women significantly underrepresented despite achieving equal medical school graduation rates. In countries like Switzerland and the United States, the gender ratio in radiology is approximately 1 woman for every 4 men.^[5]^ This imbalance raises ethical concerns regarding gender equality in medicine. Women often gravitate towards specialties perceived as more welcoming or flexible, typically avoiding fields like radiology, which are associated with high technical demands and limited patient interaction.^[6]^ Factors such as inadequate exposure to radiology, insufficient mentorship, and concerns about radiation exposure further dissuade female candidates.^[7]^

Despite its essential role in healthcare delivery and growing demand due to technological advances, radiology struggles to attract a diverse pool of medical students. Factors such as an intellectual environment, contributions to patient care, and favorable work-life balance typically draw students to the field, while worries about artificial intelligence and limited patient engagement do not significantly detract from their interest.^[8,9]^ However, there remains a scarcity of regional and institutional research on this subject, highlighting a pronounced need for further exploration.

This study aims to investigate the specific factors influencing medical students at King Khalid University (KKU) in their decision to specialize in radiology. By examining these determinants, the research seeks to provide insights that can inform radiology training programs and strategies, promoting a more equitable distribution of healthcare professionals in Saudi Arabia. Ultimately, understanding the complexities of specialty selection is essential for developing targeted interventions that enhance medical education and address the evolving needs of healthcare systems both locally and globally.

### 1.1. Aim of the study

This study aimed to investigate factors influencing the decision against or for specialty training in radiology among medical students and interns at King Khaled University, Abha, Saudi Arabia.

## 2. Methodology

### 2.1. Study design and setting

A cross-sectional study was conducted using stratified random sampling to gather data from a representative sample of students across different academic years. The study was conducted between July and December 2024. We hypothesized that there are significant factors influencing the decision of medical students and interns at KKU, Abha, Saudi Arabia, either in favor of or against pursuing specialty training in radiology. These factors may include perceived job prospects, interest in the field, lifestyle considerations, mentorship experiences, and awareness of the training requirements. The survey explored various factors such as demographics, educational experiences, and perceptions of radiology as a career using a self-administered questionnaire.

### 2.2. Data collection tools

A self-constructed questionnaire was utilized for data collection. It consisted of closed-ended questions. The questionnaire was developed by the researchers based on the literature review and further reviewed by experts for validity and applicability. A pilot study was conducted, which consisted of 30 questionnaires. This initial data was analyzed but not included in the results. The questionnaire was then modified and validated for data collection. Data was collected by the principal investigator after taking informed verbal consent and ensuring students that their identities were kept confidential. Informed consent was obtained, and participant anonymity was ensured to maintain ethical standards.

### 2.3. Participants inclusion and exclusion criteria

The inclusion criteria consisted of male and female medical students and interns at KKU who agreed to participate. Exclusion criteria included nonmedical students, health-related students from other universities, and incomplete questionnaires. The study received ethical approval from the Research Ethics Committee at KKU (HAPO-06-B-001) under approval no. ECM#2024-1214.

#### 2.3.1. Study size

The sample size was determined using the Raosoft sample size calculator (Raosoft.com [2015], Raosoft, Inc., Seattle, WA), yielding a total of 377 participants. This calculation was based on a margin of error of 5%, a 95% confidence interval, and an assumed response distribution of 50%.

### 2.4. Statistical analysis plan

Data entry for the study was performed using the Microsoft Excel program. Subsequently, the data were transferred to the Statistical Package of Social Science Software (SPSS) program, version 23.0 (TBM SPSS Statistics for Windows, Version 23.0. Armonk, NY: IBM Corp.) for statistical analysis. Categorical variables were presented as percentages and frequencies, and numerical data were presented as means and standard deviations. The Chi-square test and Fisher’s exact test were used for the association between categorical variables. Statistical significance was set at a *P*-value <.05.

## 3. Results

A total of 380 medical students and interns at KKU were included in the current study. Males outnumbered females (55.3% vs. 44.7%, respectively). Nearly two-thirds of the participants (n = 239, 62.9%) belonged to the age group of 22 to 25 years, and 125 (32.9%) were in the age group of 18 to 21 years. Most of the participants (41.8%) thought that the amount of exposure to radiology as medical students was too little, while 142 (37.4%) believed it was just right. More than half of the participants (n = 212, 55.8%) had both medical and surgical interests, and about 171 (45%) intended to pursue a particular specialty at that stage (Table 1).

**Table 1:** Characteristics of the study participants (n = 380).

Variable	Categories	N (%)
Gender	Male	210 (55.3)
	Female	170 (44.7)
Age (in years)	18–21	125 (32.9)
	22–25	239 (62.9)
	26–29	14 (3.7)
	≥ 30	2 (0.5)
Do you think the amount of exposure to radiology as medical student is:	Just right	142 (37.4)
	Too little	159 (41.8)
	Too much	79 (20.8)
Is your interest surgical, medical or both?	Medical	96 (25.3)
	Surgical	72 (18.9)
	Both	212 (55.8)
Are you intending to pursue a particular specialty at this stage?	Yes	171 (45)
	No	63 (16.6)
	Maybe	146 (38.4)

N = number, % = percentage.

Regarding the specialty preferences, the most selected specialty by the participating medical students and interns as first preference was surgery, which was selected by 39 (10.3%) of the participants as their first preference, followed by anesthesia (9.5%), radiology (9.2%), pediatrics (8.4%), and other specialties (7.9%). When the participants were asked to order the specialties from their first preference to the last preference, the overall means revealed that “other than the mentioned specialties” was the most preferred followed by surgery, pediatrics, radiology, and then emergency medicine (Table 2).

**Table 2:** Specialty preferences among participants.

Specialty	The specialty selected as first preference N (%)	Mean ± SD	Rank
Anesthesia	36 (9.5)	3.32 ± 2.39	13
Emergency medicine	25 (6.6)	3.02 ± 1.64	5
General practice	15 (3.9)	3.30 ± 1.62	12
Medicine (cardiology)	28 (7.4)	3.11 ± 1.59	7
Medicine (gastroenterology)	22 (5.8)	3.07 ± 1.35	6
Medicine (neurology)	15 (3.9)	3.48 ± 1.70	14
Medicine (respiratory)	23 (6.1)	3.13 ± 1.53	8
Obstetrics and gynecology	19 (5)	3.24 ± 1.86	9
Pediatrics	32 (8.4)	2.94 ± 1.59	3
Pathology	16 (4.2)	3.29 ± 1.97	11
Psychiatry	11 (2.9)	3.26 ± 1.63	10
Radiology	35 (9.2)	2.99 ± 2.02	4
Surgery	39 (10.3)	2.82 ± 2.22	2
Others	30 (7.9)	2.19 ± 1.57	1

N = number, SD = standard deviation, % = percentage.

**Figure 1.**
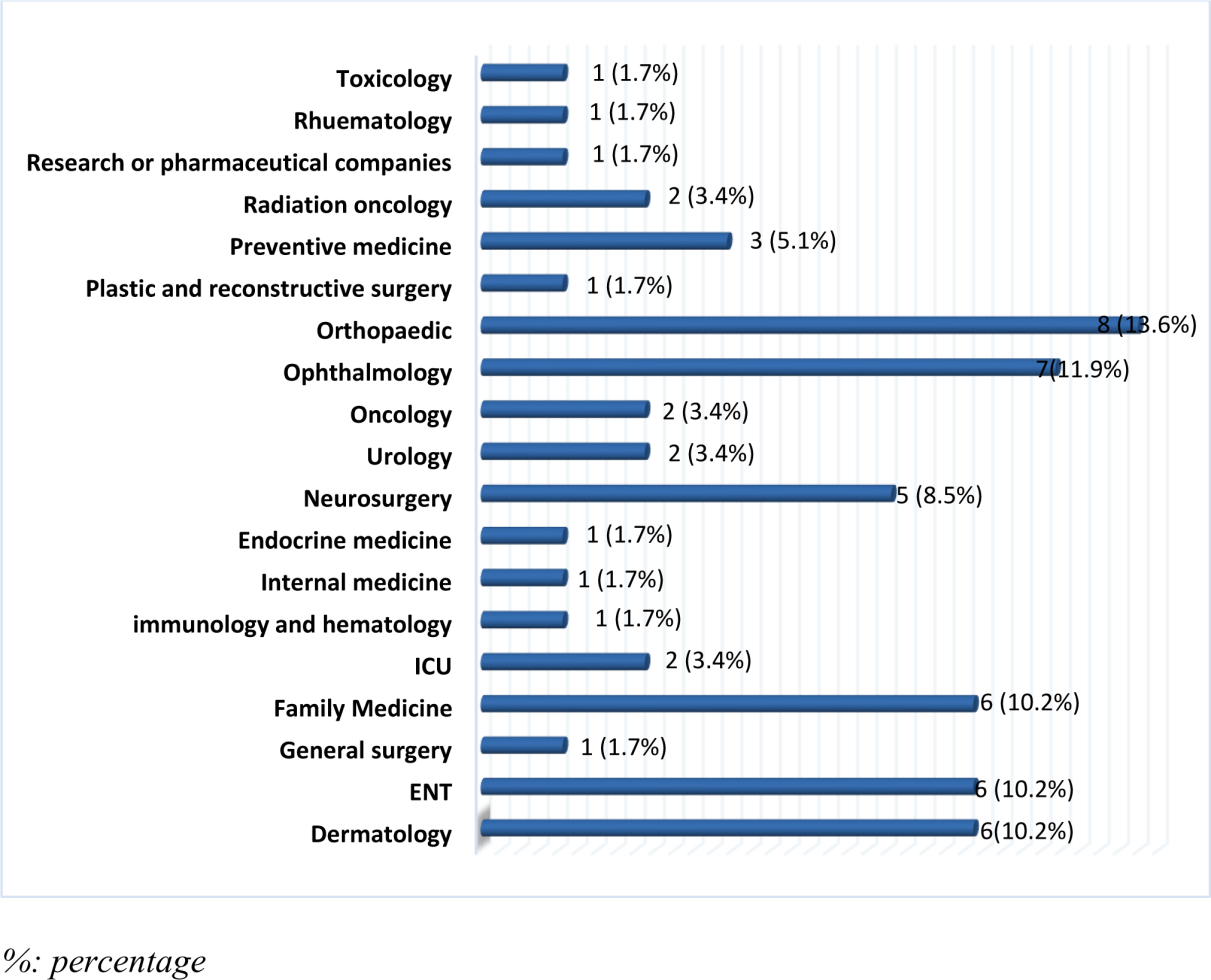
Distribution across various specialties (n = 59).

Among the other specialties mentioned by the participants, orthopedics was the most reported 1 (13.6%), followed by ophthalmology (11.9%), and then family medicine, ear, nose and throat, and dermatology (10.6%) for each) (Fig. 1).

Regarding the factors that influenced the choice of specialty, more than a third of the participants thought that academic examination results and competition for training schemes had a strong influence on their choice of specialty (38.2% and 35.3% respectively), while a higher percentage believed that diversity within the field and the extent of patient contact strongly influenced their decision (43.4% and 50.8% respectively). Family expectations were reported by 109 (28.7%) of the participants as a strong influencer on their specialty choice. Most of the participants believed that income potential and future earnings, interest in acute patient management, and current exposure or knowledge in the field strongly influenced their decision, representing 171 (45%), 159 (41.8%), and 157 (41.3%) of the participants, respectively. Nearly a third of the participants thought that the length of training and work/life balance strongly affected their selection of specialty (30.3% and 29.7%, respectively). Moreover, most participants believed that the status of the specialty, the influence of mentors or role models, potential litigation and legal issues, and the working environment and relationships had a strong influence on their choice of specialty, accounting for 46.1%, 36.6%, 32.1%, and 30.3% respectively (Table 3).

**Table 3:** Factors influencing the choice of specialty.

Factors:	Strong influence	Moderate influence	Minor influence	No influence
	N (%)			
Academic examination results	145 (38.2)	72 (18.9)	53 (13.9)	110 (28.9)
Competition for training scheme	134 (35.3)	104 (27.4)	101 (26.6)	41 (10.8)
Diversity within the field	165 (43.4)	100 (26.3)	68 (17.9)	47 (12.4)
Extent of patient contact	155 (40.8)	86 (22.6)	99 (26.1)	40 (10.5)
Family expectations	109 (28.7)	94 (24.7)	92 (24.2)	85 (22.4)
Income potential and future earnings	171 (45)	97 (25.5)	73 (19.2)	39 (10.3)
Interest in acute patient management	159 (41.8)	83 (21.8)	67 (17.6)	71 (18.7)
Current exposure/ knowledge in the field	157 (41.3)	88 (23.2)	100 (26.3)	35 (9.2)
Length of the training	115 (30.3)	104 (27.4)	104 (27.4)	57 (15)
Work/life balance	113 (29.7)	80 (21.1)	152 (40)	35 (9.2)
Mentor or role model influence	139 (36.6)	85 (22.4)	97 (25.5)	59 (15.5)
Potential litigation and legal issues	122 (32.1)	111 (29.2)	87 (22.9)	60 (15.8)
Status of the specialty	175 (46.1)	96 (25.3)	71 (18.7)	38 (10)
Working environment & relationships	115 (30.3)	78 (20.5)	73 (19.2)	114 (30)

N = number, % = percentage.

Concerning the exposure of participating medical students and interns to radiology, about 239 (62.9%) of the participants had exposure to radiology. In detail, 173 (45.5%) of the participants were exposed to the stage 1 healthcare imaging and information system module, and 148 (38.9%) were exposed to the radiology and diagnostic imaging option module. Less than a third of the participants were exposed to other core module(s) and other elective module(s) (29.5% and 22.9% respectively). About 112 (29.5%) of the participants had a previous qualification in radiology, 136 (35.8%) had previous clinical experience related to radiology, and 169 (44.5%) had previous personal experience related to radiology (Table 4)

**Table 4:** Exposure to radiology.

Question	Yes	No
	N (%)	
To date have you had any exposure to radiology?	239 (62.9)	141 (37.1)
What is your exposure to radiology?		
Stage 1 healthcare imagining and information system module	173 (45.5)	207 (54.5)
Radiology & diagnostic imaging option module	148 (38.9)	232 (61.1)
Other core module(s)	112 (29.5)	268 (70.5)
Other elective module(s)	87 (22.9)	293 (77.1)
Previous qualification	112 (29.5)	268 (70.5)
Previous clinical experience related to radiology	136 (35.8)	244 (64.2)
Previous personal experience related radiology	169 (44.5)	211 (55.5)

N = number, % = percentage.

**Figure 2.**
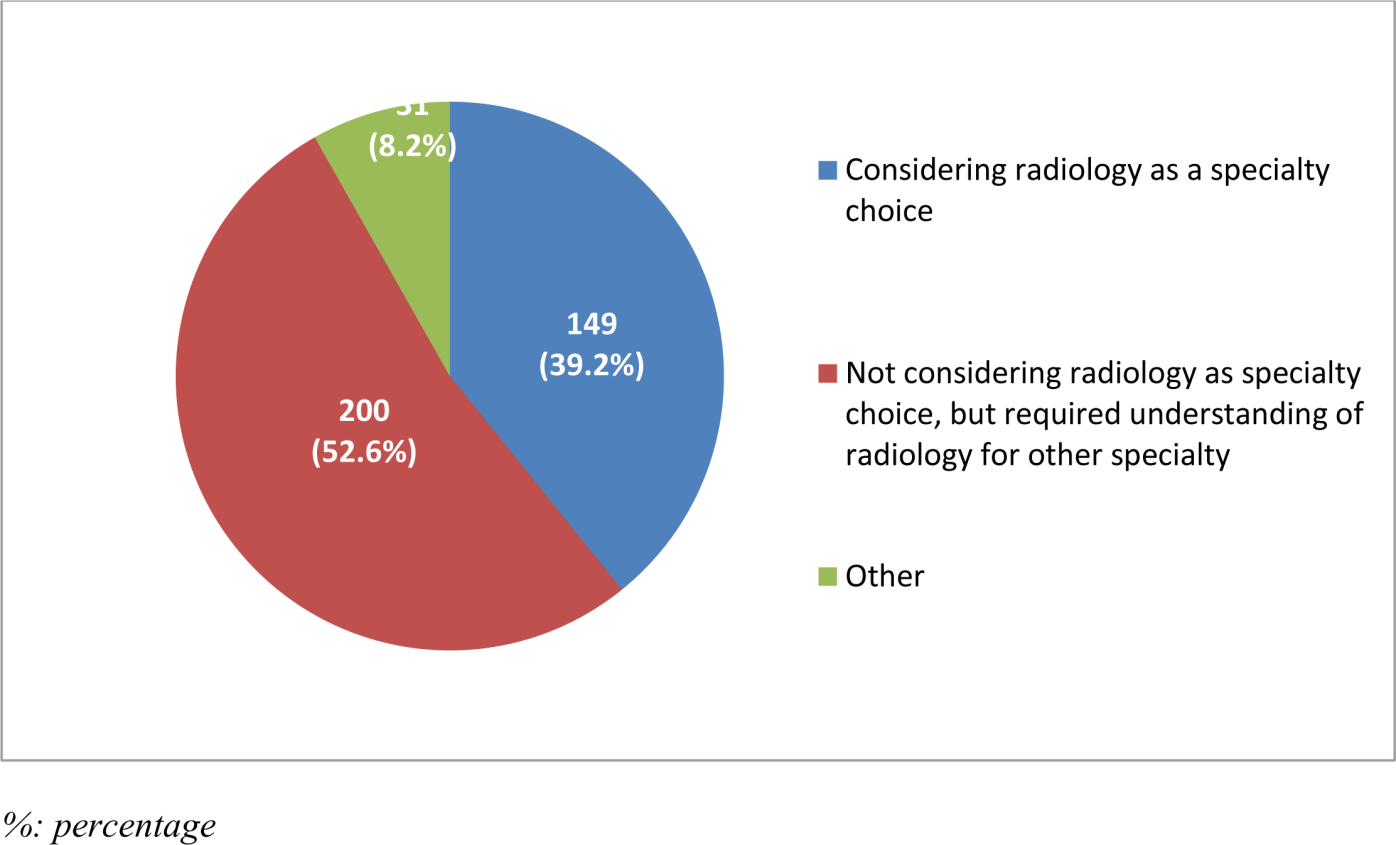
Reasons for choosing to take a radiology course.

When the participants were asked why they chose to take the radiology course or experience, more than half of them (n = 200, 52.6%) said that they did not consider radiology as a specialty choice, but they needed to understand it for another specialty, while 149 (39.2%) said that they considered radiology as a specialty choice (Fig. 2).

**Figure 3.**
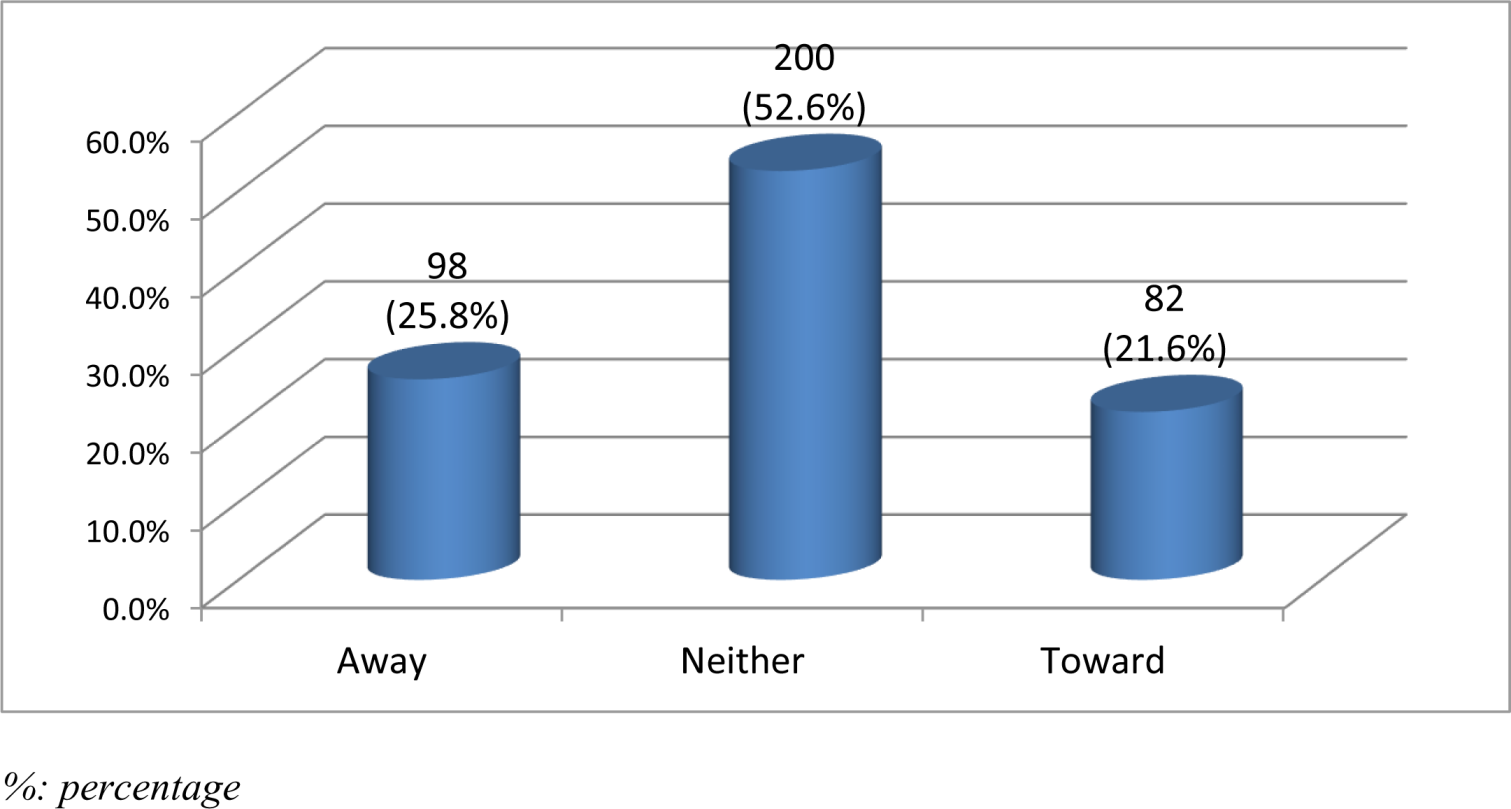
Impact of radiology exposure on specialty choice: attraction or deterrence.

Concerning the effect of exposure to radiology, more than half of the participants (n = 200, 52.6%) neither turned them toward nor away from radiology as a specialty, while 98 students (25.8%) reported that their exposure turned them away from the radiology specialty (Fig. 3).

Regarding the responsibilities of radiologists, the vast majority of the participants (n = 343, 90.3%) believed that radiologists diagnosed patients’ conditions. About 148 (38.9%) of the participants thought that radiologists performed direct medical treatment, while 60.8% believed that radiologists carried out direct surgical treatment. Nearly one third of the participants (32.4%) thought that radiologists met with patients. The majority of the participants believed that radiologists had other responsibilities, such as performing medical and surgical procedures (64.5% and 61.1%, respectively). Additionally, some mentioned that radiologists could conduct conventional radiographic examinations (65.0%), optimize parameters used for different imaging modalities (61.1%), and ensure patient safety by minimizing radiation exposure while maintaining diagnostic image quality (Table 5).

**Table 5:** Participants’ knowledge about the responsibilities of radiologists.

Responsibilities	Yes	No
	N (%)	
Diagnosis patient	343 (90.3)	37 (9.7)
Direct medical treatment	148 (38.9)	232 (61.1)
Direct surgical treatment	231 (60.8)	149 (39.2)
Meet the patient	123 (32.4)	257 (67.6)
Perform medical procedure	245 (64.5)	135 (35.5)
Perform surgical procedure	232 (61.1)	148 (38.9)
Perform conventional X-rays/ radiographic examinations e.g. chest and musculoskeletal X-ray	247 (65.0)	133 (35)
Direct radiographers to optimize/set the parameters used for differing image modalities	232 (61.1)	148 (38.9)
Ensure patient safety by minimizing radiation exposure while maintaining diagnostic image quality	245 (64.5)	135 (35.5)

CT = computed tomography, N = number, % = percentage.

**Figure 4.**
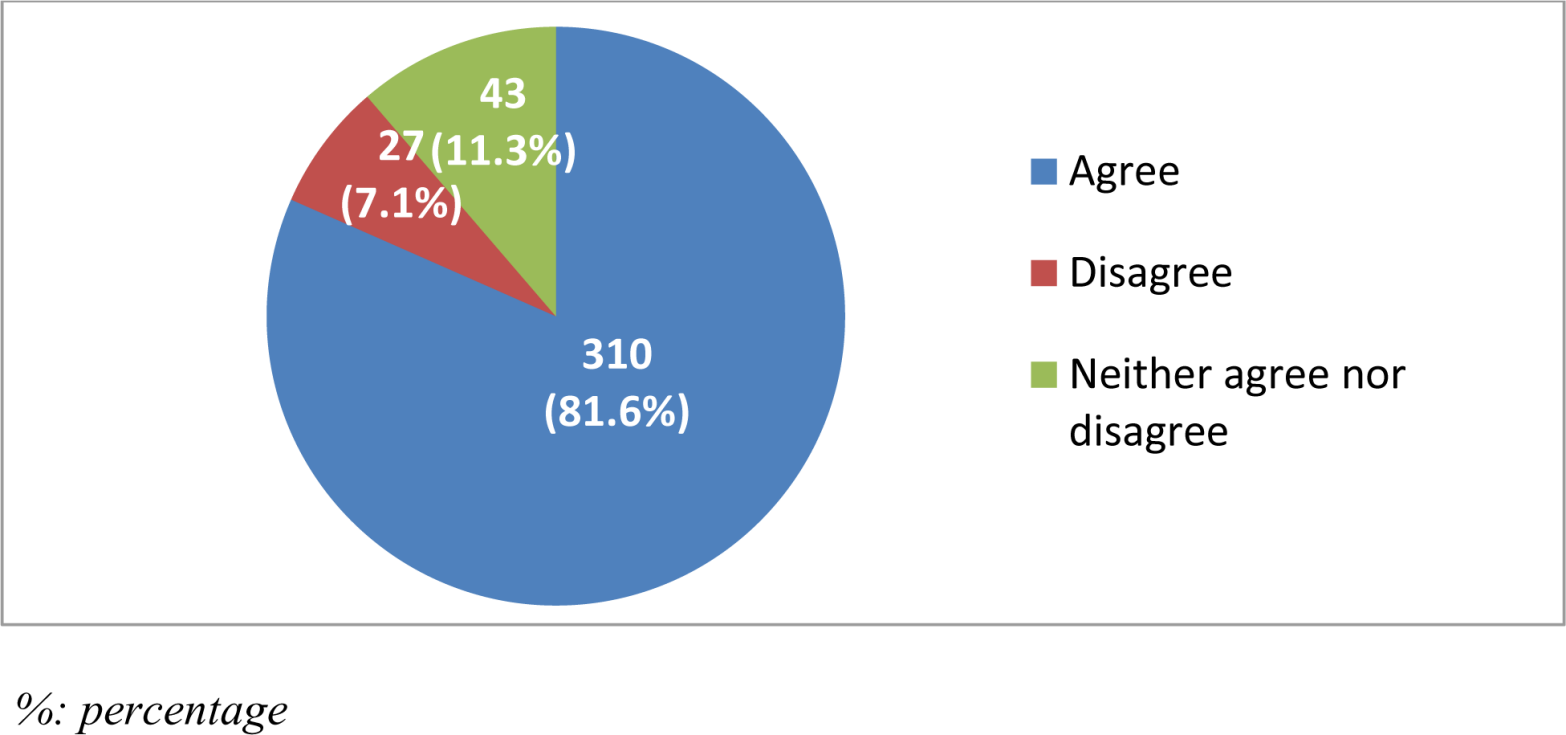
Radiologists play an important role in diagnosis and patient management.

Regarding the role of radiologists in diagnosis and patient management, the majority (n = 310, 81.6%) believed that radiologists played an important role, while 27 (7.1%) disagreed and 43 (11.3%) were neutral (Fig. 4).

**Figure 5.**
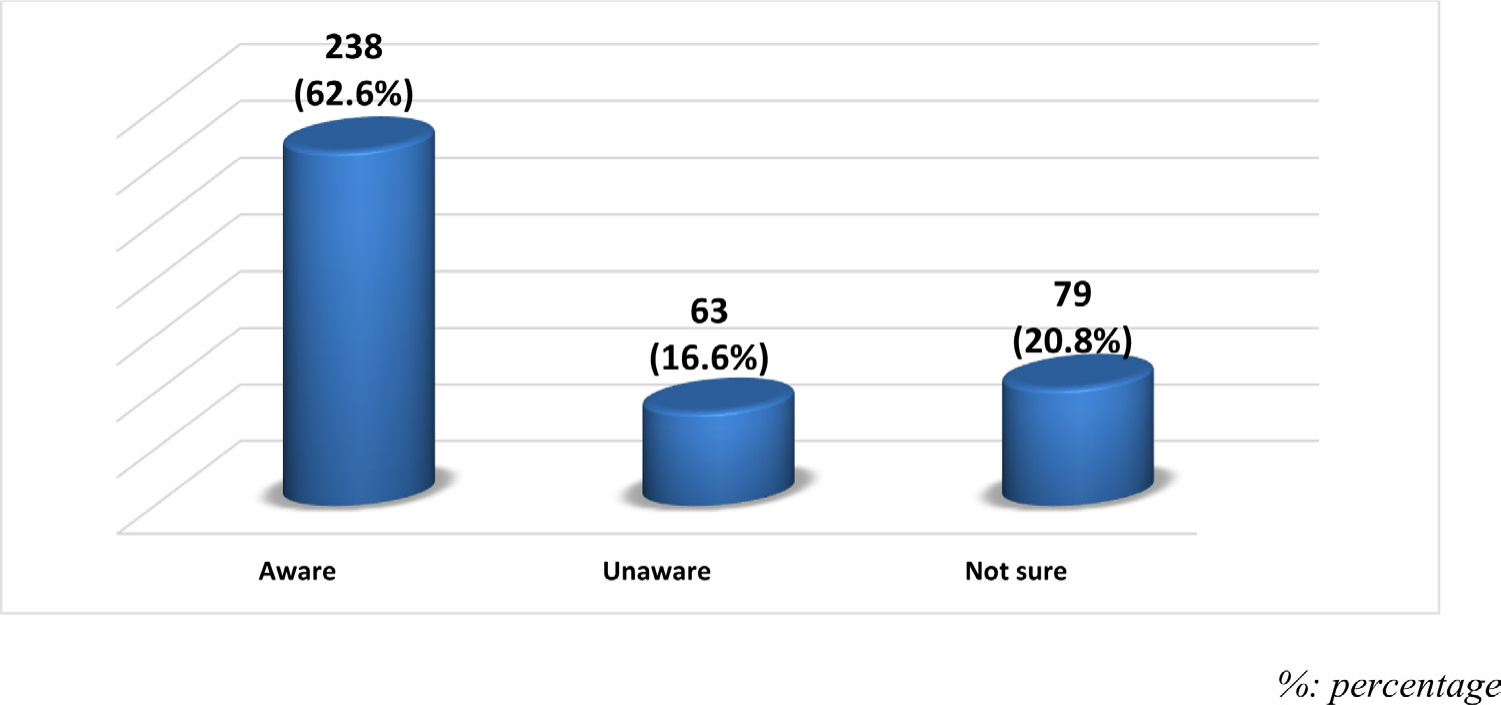
Participants’ awareness about the difference between a radiologist and a radiographer.

Regarding participants awareness about the difference between a radiologist and a radiographer, about 238 (62.6%) of the participants were aware of the difference between them, while 63 (16.6%) were not aware and 79 (20.8%) were not sure (Fig. 5).

**Figure 6.**
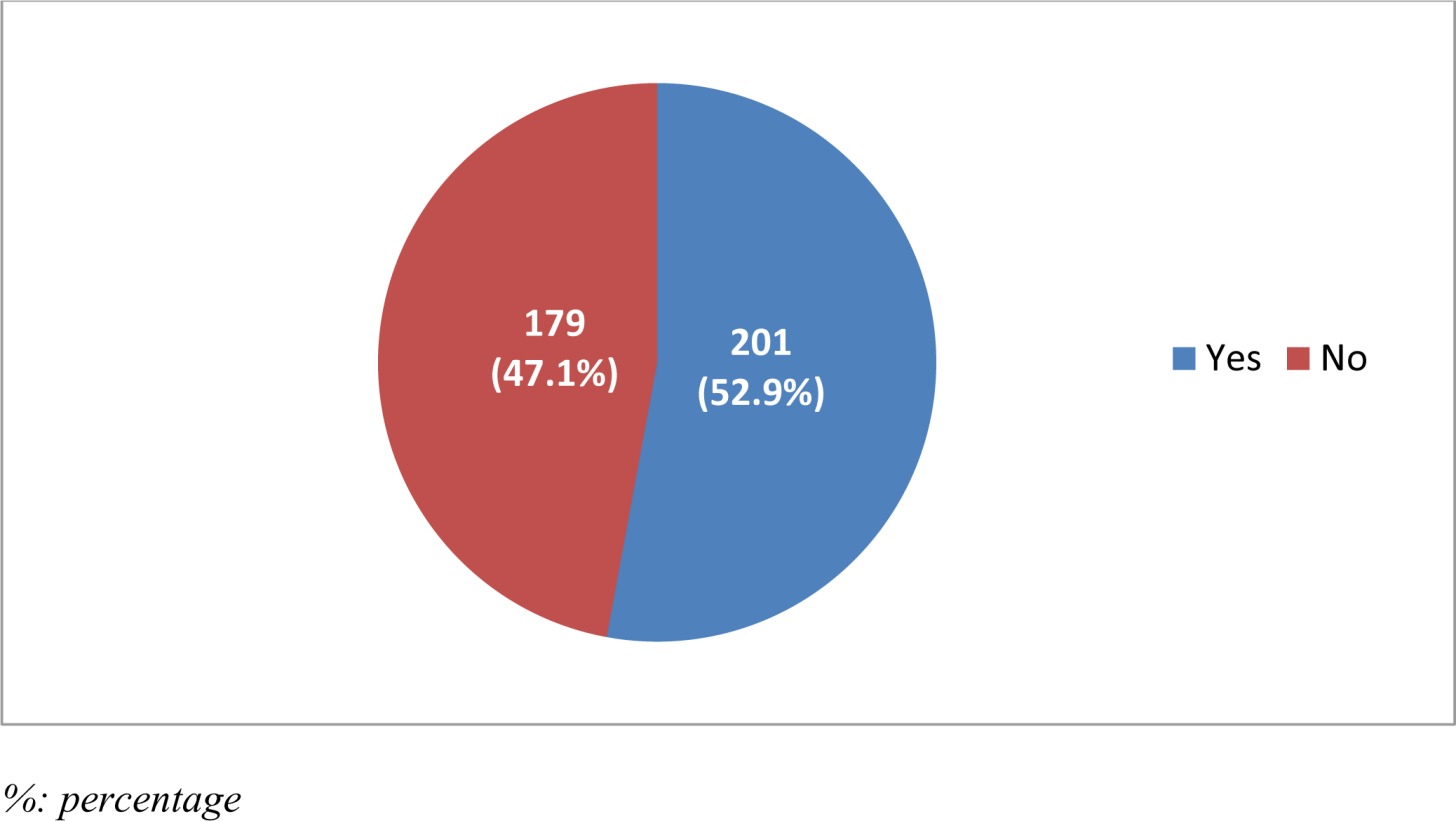
Consideration of radiology as a future specialty.

More than half of the participants (n = 201, 52.9%) considered radiology as a future specialty, while 179 (47.1%) did not (Fig. 6).

Regarding the reasons for considering radiology and reasons for not considering radiology, the most reported reasons for considering radiology were variation of the specialty (68.2%), the appealing aspects of radiology, like its diversity and content (63.4%), and minimal patient interaction (63.4%), while the most reported reasons for not considering radiology were the risk of radiation exposure (78.7%), deciding to pursue another specialty (53.4%), and too little patient interaction (47.6%) (Table 6).

**Table 6:** Reasons for considering radiology and reasons for not considering radiology.

Reasons for considering radiology	N (%)	Reasons for not considering radiology	N (%)
Very interested in the specialty	189 (49.7)	Interesting but do not want to do all the time	172 (45.3)
Physics/technology	138 (36.3)	Physics/technology component not appealing	142 (37.4)
Appealing aspects of radiology, like its diversity and content	241 (63.4)	Too little patient interaction	181 (47.6)
Minimal patient interaction	241 (63.4)	Too much patient interaction	117 (30.8)
Lots of patient interaction	213 (56.1)	Risk of radiation exposure	299 (78.7)
Risk of radiation exposure	135 (35.5)	My mind is made up to pursue another specialty	203 (53.4)
Radiology training is competitive	124 (32.6)	Radiology training is too competitive	143 (37.6)
Radiology is very varied	259 (68.2)	Radiology would not be varied enough	150 (39.5)
I know somebody working in radiology	162 (42.6)	I know somebody working in radiology	126 (33.2)

N = number, % = percentage.

Regarding the association between considering radiology as a future specialty and other factors, the results revealed that there were significant associations between prior exposure to radiology, the effect of exposure to radiology, and knowing the difference between a radiologist and a radiographer in relation to selecting radiology as a future specialty (*P* < .001 for all). Participants who were previously exposed to radiology indicated that this turned them toward radiology as a specialty, and participants who knew the difference between a radiologist and a radiographer were more likely to choose radiology as a future specialty than others. “Other factors, including gender, age, duration of exposure to radiology, and participant interest, were not significantly associated with the decision to pursue radiology as a future specialty (*P* > .05). (Table 7).

**Table 7:** Factors influencing considering radiology as a future specialty.

Variable	Categories	Are you considering radiology as a future specialty?		P value
		Yes	No	
		N (%)		
Gender	Male	118 (56.2)	92 (43.8)	.153^C^
	Female	83 (48.8)	87 (51.2)	
Age (in years)	18–21	75 (60)	50 (40)	.055^F^
	22–25	121 (50.6)	118 (49.4)	
	26–29	5 (35.7)	9 (64.3)	
	≥ 30	0 (0)	2 (100)	
Do you think the amount of exposure to radiology as medical student is:	Just right	80 (56.3)	62 (43.7)	.333^C^
	Too little	77 (48.4)	82 (51.6)	
	Too much	44 (55.7)	35 (44.3)	
Is your interest surgical, medical or both?	Medical	42 (43.8)	54 (56.3)	.109^C^
	Surgical	39 (54.2)	33 (45.8)	
	Both	120 (56.6)	92 (43.4)	
Previous exposure to Radiology	Yes	145 (60.7)	94 (39.3)	< .001^C^
	No	56 (39.7)	85 (60.3)	
Did this exposure to radiology affect your choice for radiology as a specialty?	Away	28 (28.6)	70 (71.4)	< .001^C^
	Neither	98 (49)	102 (51)	
	Toward	75 (91.5)	7 (8.5)	
Do you know the difference between a radiologist and a radiographer?	Yes	143 (60.1)	95 (39.9)	.001^C^
	No	27 (42.9)	36 (57.1)	
	Not sure	31 (39.2)	48 (60.8)	

C = Chi-square test, F = Fisher’s exact test, N = number, % = percentage.

## 4. Discussion

The expansion of imaging technologies has significantly augmented the scope of radiology, rendering it an indispensable medical specialty. However, the decision to pursue radiology is influenced by multiple factors, including personal interest, lifestyle considerations, and perceived workload. This study aimed to explore the determinants affecting medical students and interns at KKU in Abha, Saudi Arabia, regarding their specialty choices.

### 4.1. Demographic overview

The participant demographics revealed a fairly balanced gender ratio (55.3% male, 44.7% female), with the majority of participants aged 22–25 years (62.9%). This pattern is consistent with previous research, which suggests that final-year medical students are more focused on specialty preferences and career decisions (Querido et al., 2018).^[1]^ However, a concerning 41.8% of participants felt their exposure to radiology was inadequate, which aligns with findings from other studies linking limited exposure during medical training to a reduced interest in radiology as a specialty.^[2,10]^

### 4.2. Specialty preferences

When it comes to specialty preferences, surgery was the most favored choice, followed by anesthesia, radiology, and pediatrics. This pattern contrasts with trends observed in Sudan, where radiology ranks higher due to its favorable work-life balance and technological appeal.^[3]^ Orthopedic surgery (13.6%) was particularly favored for its hands-on nature and high earning potential,^[5]^ and ophthalmology (11.9%) saw increasing interest, as highlighted by AlSalman et al. (2017).^[6]^ Additionally, family medicine, ear, nose and throat, and dermatology each received 10.2% of participants’ preferences, reflecting the growing demand for primary care practitioners in Saudi Arabia.^[7]^

### 4.3. Factors influencing specialty choice

Several factors were identified as influencing specialty selection, such as academic performance (38.2%), competition for training positions (35.3%), and extent of patient contact (50.8%).

These findings echo prior studies emphasizing the competitive nature of medical training and the significant impact of academic results on career decisions.^[11]^ While preferences for specialties based on patient interaction varied, the influence of work-life balance (29.7%) and training duration (30.3%) appeared less significant in our sample. This contrasts with other studies where these factors were highlighted as major considerations.^[12–15]^

### 4.4. Exposure to radiology

Exposure to radiology during training is crucial in sparking interest in the field. In this study, 62.9% of participants had exposure to radiology, which is essential, as early exposure has been shown to increase interest in the specialty (Pape et al., 2023).^[16]^ Moreover, 35.8% had prior clinical experience related to radiology, and 44.5% had personal experience, figures higher than those reported in a systematic review by Bwanga and Lidster (2019).^[17]^ These results emphasize the need for more clinical engagement opportunities through elective modules, as suggested by Tahir et al. (2024).^[18]^

Interestingly, 52.6% of participants took a radiology course not with the intent to pursue it as a specialty but to gain knowledge useful in other fields. This underscores radiology’s role as a supplementary knowledge base across various medical domains.^[19]^ Additionally, 39.2% of participants considered radiology as a potential specialty, a figure higher than typically reported internationally.^[20,21]^ This could be attributed to increased exposure at KKU and the rising demand for radiologists in Saudi Arabia. Despite this, 52.6% reported that their exposure neither encouraged nor discouraged them from pursuing radiology, suggesting the need for more impactful rotations, as supported by other studies.^[22–25]^

### 4.5. Understanding of radiology roles

There were noticeable gaps in students’ understanding of the roles of radiologists. While 90.3% recognized that radiologists have a diagnostic role, a significant percentage (60.8%) mistakenly believed that radiologists perform direct surgical treatments. However, there was solid awareness of radiologists’ technical responsibilities, such as conducting conventional X-rays (65%) and ensuring radiation protection (64.5%).^[2]^

### 4.6. Motivations and barriers

While 52.9% of participants expressed interest in pursuing radiology as a future specialty—an encouraging figure exceeding international averages of 20–40%^[3,18]^—this finding warrants further exploration. The dual perceptions regarding radiology’s diversity (68.2%) and engaging content (63.4%) suggest that students recognize its intellectually stimulating nature; however, the fact that an equal proportion (63.4%) also cited minimal patient interaction as a positive aspect may reflect a preference for specialized, less patient-dependent roles. Conversely, nearly half of the participants (47.6%) viewed the limited patient contact as a deterrent, indicating a potential area for addressing perceptions about the scope of radiology practice. Additionally, concerns surrounding radiation exposure (78.7%) remain prevalent,^[12]^ underscoring ongoing safety considerations that may influence specialty choice. These insights highlight the complex interplay of motivation and apprehension shaping students’ career preferences and suggest targeted interventions could influence future interest levels.

### 4.7. Associations and analysis

Significant associations were found between students’ exposure to radiology, understanding of the difference between radiologists and radiographers, and their consideration of radiology as a specialty (*P* < .001 for all). This reinforces the positive impact of targeted educational experiences in increasing interest in the specialty.^[19,26]^ Interestingly, gender and age did not significantly influence specialty interest, suggesting that regional factors in Saudi Arabia may differ from findings in other regions.^[16,27]^

### 4.8. Study limitations

This study has some limitations. Its focus on a single institution may limit the generalizability of the findings to other medical schools in Saudi Arabia or internationally. Additionally, the cross-sectional design provides only a snapshot of students’ perceptions, potentially overlooking changes in their views over time. Lastly, the reliance on self-reported data may introduce response bias, which could affect the authenticity of the participants’ responses.

## 5. Conclusion

This study highlights the factors influencing specialty choices among medical students and interns at KKU. Key factors such as academic performance, patient contact, and personal preferences shape students’ career decisions. The study found that 39.2% of participants expressed interest in radiology, with 62.9% having some exposure to the field. The appeal of radiology is largely due to its diversity and minimal patient interaction, making it an attractive option for students seeking engaging and manageable career paths in medicine. Barriers to pursuing radiology include concerns about radiation exposure and a preference for other specialties. The study found a strong correlation between exposure to radiology and the likelihood of choosing it as a specialty.

This emphasizes the importance of enhancing educational exposure to the field. Increasing opportunities for hands-on experiences, comprehensive radiology training, and mentorship can help foster greater interest in radiology. To cultivate a new generation of radiologists, it is essential to address misconceptions surrounding radiation safety and to promote the diverse and fulfilling career opportunities within the field. By refining curricula, implementing targeted outreach, and offering more impactful educational experiences, medical schools can inspire more students to consider radiology as a dynamic and viable career path. Such efforts will ultimately contribute to strengthening the healthcare workforce and the radiology specialty.

## Acknowledgments

The authors extend their appreciation to the Deanship of Scientific Research at King Khalid University for funding this work through large group Research Project under grant number RGP.2/597/45

## Author contributions

**Conceptualization:** Hassan Ali AlMubarak

**Data curation:** Hassan Ali AlMubarak

**Investigation:** Hassan Ali AlMubarak, Abdulrahman Ali H Al Mesmer Almaymoni, Waad Saber Z Alharethi, Ohoud Hussain M Moalwi, Lamyaa Safar A Almathahibi, Reema Saleh A Alqahtani, Ahmed Ali Khuzayyim, Hanan Delem M Almoghamer, Fatimah Ahmed H Badawi, Wajan Ahmed A. Alshahrani, Lujain Adel A Daghriri, Khalid Abdulrahman A Almehery

**Methodology:** Hassan Ali AlMubarak, Abdulrahman Ali H Al Mesmer Almaymoni

**Project administration:** Hassan Ali AlMubarak

**Supervision:** Hassan Ali AlMubarak, Awad Alsamghan

**Writing—original draft:** Hassan Ali AlMubarak, Abdulrahman Ali H Al Mesmer Almaymoni

**Validation:** Awad Alsamghan

**Visualization:** Awad Alsamghan

**Writing—review & editing:** Awad Alsamghan, Syed Esam Mahmood
